# Microbial Environment Affects Innate Immunity in Two Closely Related Earthworm Species *Eisenia andrei* and *Eisenia fetida*

**DOI:** 10.1371/journal.pone.0079257

**Published:** 2013-11-01

**Authors:** Jiří Dvořák, Veronika Mančíková, Václav Pižl, Dana Elhottová, Marcela Šilerová, Radka Roubalová, František Škanta, Petra Procházková, Martin Bilej

**Affiliations:** 1 Laboratory of Cellular and Molecular Immunology, Institute of Microbiology of the Academy of Sciences of the Czech Republic, v. v. i., Prague, Czech Republic; 2 Department of Soil Zoology and Soil Microstructure, Institute of Soil Biology, Biology Center of the Academy of Sciences of the Czech Republic, v. v. i., České Budějovice, Czech Republic; University of North Carolina at Chapel Hill, United States of America

## Abstract

Survival of earthworms in the environment depends on their ability to recognize and eliminate potential pathogens. This work is aimed to compare the innate defense mechanisms of two closely related earthworm species, *Eisenia andrei* and *Eisenia fetida*, that inhabit substantially different ecological niches. While *E. andrei* lives in a compost and manure, *E. fetida* can be found in the litter layer in forests. Therefore, the influence of environment-specific microbiota on the immune response of both species was followed. Firstly, a reliable method to discern between *E. andrei* and *E. fetida* based on species-specific primers for cytochrome c oxidase I (COI) and stringent PCR conditions was developed. Secondly, to analyze the immunological profile in both earthworm species, the activity and expression of lysozyme, pattern recognition protein CCF, and antimicrobial proteins with hemolytic function, fetidin and lysenins, have been assessed. Whereas, CCF and lysozyme showed only slight differences in the expression and activity, fetidin/lysenins expression as well as the hemolytic activity was considerably higher in *E. andrei* as compared to *E. fetida*. The expression of fetidin/lysenins in *E. fetida* was not affected upon the challenge with compost microbiota, suggesting more substantial changes in the regulation of the gene expression. Genomic DNA analyses revealed significantly higher level of fetidin/lysenins (determined using universal primer pairs) in *E. andrei* compared to *E. fetida*. It can be hypothesized that *E. andrei* colonizing compost as a new habitat acquired an evolutionary selection advantage resulting in a higher expression of antimicrobial proteins.

## Introduction

The earthworms belonging to oligochaete annelids are widely used in vermicomposting and ecotoxicology. In addition, earthworms are regarded as a model of comparative biochemistry, physiology, and last but not least, immunology since early sixties when transplantation experiments proving the existence of self/nonself recognition were performed [1,2]. The earthworms possess a large variety of defense mechanisms including phagocytosis, encapsulation, and pattern recognition followed by the synthesis and secretion of antimicrobial proteins that efficiently protect themselves against infectious agents. Numerous immunologically important proteins have been characterized and cloned in earthworms. We have chosen following proteins in the present study. (i) Lysozyme, an evolutionary conserved protein that hydrolyzes bonds between N-acetylglucosamine and N-acetylmuramic acid of the peptidoglycan present in bacterial walls of Gram-positive bacteria [3,4]. (ii) Many research groups observed different types of antibacterial factors with hemolytic activity in *Eisenia fetida andrei* without clear consensus in their nomenclature and relationship. French authors described two glycoproteins secreted into the coelomic cavity [5] and later on, nucleotide sequences of these factors have been described and proteins were named fetidins [6]. Independently, hemolytic protein causing the contraction of rat vascular smooth muscles was characterized and named lysenin [7]. These hemolytic proteins play also a role as opsonins that render bacteria susceptible to phagocytosis. (iii) Last but not least, a pattern recognition protein with a cytolytic activity named coelomic cytolytic factor (CCF) was studied [8]. CCF possesses two distinct carbohydrate recognition domains that allow binding of lipopolysaccharides, β-1,3-glucans, peptidoglycan constituents, and N-acetylglycosidic bond-linked oligo- and polysaccharides [9]. Moreover, CCF mRNA level is up-regulated upon the microbial stimulation [10].

Previously, we have described that the natural environment has a significant effect on the defense mechanisms of earthworms. Comparative study of CCF in eight earthworm species inhabiting different soil horizons revealed differences in the recognition specificity of this pattern recognition molecule [11]. We have proposed that the variation of microbiota composition in the soil horizons can affect the repertoire of the defense system of earthworms. 

This study focuses on the immunological comparison of two closely related earthworm species inhabiting significantly different habitats, *Eisenia andrei* [12] and *Eisenia fetida* (Savigny, 1826). These two species were first described as different morphotypes of *E. fetida* according to differences in the body pigmentation [13], and subsequently established as subspecies [12] named *Eisenia fetida andrei* and *Eisenia fetida fetida*. Recently, they are considered as two independent species, *E. andrei* and *E. fetida* [14]. However, a reliable discrimination of these two species is rather difficult due to the variability of morphological and anatomical characteristics. It was described that nucleotide sequences of a mitochondrial gene for cytochrome c oxidase subunit I (COI) differ in these two species [15]. Thus, we designed two sets of species-specific primers, which in combination with stringent PCR conditions allowed us to easily and reliably distinguish *E. andrei* from *E. fetida*. 

Above mentioned two earthworm species share many similarities, but their natural environment substantially varies. Whereas *E. andrei* lives in a compost and manure rich in potential pathogens, non-synantropic indigenous populations of *E. fetida* earthworm can be found in the litter layer of moist forests that are considerably less abundant in a number of microorganisms [16]. Therefore, it was of interest to inquire how the natural environment and its microbiota affect various defense mechanisms of earthworms. In the present study we demonstrate the effect of compost and forest-soil microbiota on the immune mechanisms of *E. fetida* and *E. andrei* earthworms. While the gene expression and biological activities of lysozyme and CCF do not differ in both species, the gene expression of fetidin and lysenin genes as well as the hemolytic activity of the coelomic fluid of *E. andrei* is significantly higher in comparison with that of *E. fetida*. 

## Materials and Methods

### Animals, collection of the coelomic fluid, and isolation of coelomocytes

To avoid sample contamination by gut content, adult earthworms of both species (*E. andrei* and *E. fetida*) were maintained on moist paper towels for two days prior to the coelomic fluid and coelomocyte collection. Coelomic fluid containing free coelomocytes was collected by puncturing post-clitellum segments of the coelomic cavity with a Pasteur micropipette. Coelomocytes were obtained by subsequent centrifugation (10 min, 500×g, 4 °C). The supernatant, i.e. cell-free coelomic fluid was collected and re-centrifuged (10 min, 1000×g, 4 °C). Prior to use in bioassays, the protein concentration of the coelomic fluid was determined by the Lowry method (DC Protein Assay, Bio-Rad). 

### DNA isolation

Three independent genomic DNA isolations (each from 5 mg of tissue of four individuals of both *E. andrei* and *E. fetida*) were done using MasterPure complete DNA & RNA purification kit (Epicentre) according to the manufacturer’s protocol. Isolated genomic DNA was used in PCR reaction.

### RNA isolation, cDNA synthesis

Total RNA was isolated from coelomocytes and tissues (approximately 50 µg) using TRIZOL reagent (Life Technologies) according to the manufacturer’s protocol. Three independent RNA isolations each pooled from four individuals were performed. One microgram of DNAse I-treated total RNA was reverse-transcribed using Oligo(dT)_12-18_ primer and Superscript II RNase H^-^ Reverse Transcriptase (Life Technologies) and subsequently used in PCR reactions.

### PCR and Rapid Amplification of cDNA Ends (RACE)

Universal primers for both *E. andrei* and *E. fetida* cytochrome c oxidase subunit I (COI) used in the PCR are shown in the Table 1. A fragment of 541 bp was amplified using the following cycling parameters: 2 min at 94 °C, followed by 35 cycles of 30 s at 94 °C, 40 s at 56 °C and 90 s at 72 °C and a final extension for 10 min at 72 °C. The PCR product was ligated into pCR2.1-TOPO cloning vector (Life Technologies) and sequenced. 3‘ and 5‘ ends of COI cDNAs were obtained using 3‘ and 5‘ RACE System (Rapid Amplification of cDNA Ends, Life Technologies) and obtained PCR products were cloned into pCR2.1-TOPO vector (Life Technologies).

**Table 1:** Sequences of universal and species-specific primers for COI used in PCR reactions.

**Universal primers for *COI***		
COI F	*sense*	5‘-AACCAGGTGCCTTCCTAGG-3‘
COI R	*antisense*	5‘-GCAGGATCAAAGAATGAGGT-3‘
**Discriminating primers for *E. andrei COI***		
COI EA 1	*sense*	5‘-GGATTTGGAAACTGACTTC-3‘
COI EA 2	*antisense*	5‘-CCGCGTGCGCTAAGTTACTG-3‘
COI EA 3	*sense*	5‘-CCCACCCCTATCCAGTAACT-3‘
COI EA 4	*antisense*	5‘-TCGTTCTAGTCGAAGCCCAC-3‘
**Discriminating primers for *E. fetida COI***		
COI EF 1	*sense*	5‘-GGGTTTGGAAACTGATTGT-3‘
COI EF 2	*antisense*	5‘-CGGCATGTGCGAGATTAGCC-3‘
COI EF 3	*sense*	5‘-CCCCCCCTTATCGGGTAATC-3‘
COI EF 4	*antisense*	5‘-TCGCTCTAGCCGCAACCCCT-3‘

### Sequencing

Isolated and purified plasmid DNA was sequenced with ABI PRISM BigDye Terminator v3.1 Cycle sequencing Kit (Applied Biosystems). The chain termination reaction [17] was performed by cycle sequencing technique [18] according to manufacturer’s protocol. Finally, sequences were determined using an ABI PRISM 3100 DNA sequencer (Applied Biosystems) and whole coding sequences were submitted to the GeneBank databasis (*E. andrei* COI – NCBI: HQ534065, *E. fetida* COI – NCBI: HQ534066).

### PCR discriminating between *E. andrei* and *E. fetida*

Based on the retrieved complete nucleotide sequences of COI species-discriminating (*E. andrei* and *E. fetida*) primers were designed. Primer sequences are listed in the Table 1. Combinations of primers were used in PCR reaction as follows COI 1/COI 2, COI 1/COI 4, COI 3/COI 4. Cycling conditions were: 2 min at 94 °C, followed by 34 cycles of 30 s at 94°C, 40 s at 62,5 °C and 90 s at 72 °C and a final extension for 10 min at 72 °C. 

### Hemolytic assay

Hemolytic activity of the coelomic fluid was tested in 96-well microtiter plates (type V). A sample of 100 μl of *E. andrei* or *E. fetida* serially diluted coelomic fluid with inhibitor of serine proteases CompleteTM (Roche) was diluted in 145 mM NaCl (pH 7.4) and then mixed with 100 μl of sheep erythrocyte suspension (3% in 145 mM NaCl, ph 7,4) and incubated for 2 hours at room temperature. The plates were centrifuged (10 min, 100×g, 4 °C) and the absorbance of supernatants was measured at 405 nm. The percentage of hemolysis was determined using linear regression. The hemolytic assay was done in duplicates and repeated in three independent experiments.

### Cytolytic assay

Cytolytic activity was quantified using a cell-killing bioassay as described previously [8]. Murine TNF-sensitive L929 fibrosarcoma cells were cultivated in RPMI-1640 medium supplemented with 10% fetal bovine serum, 2 mM glutamine, 1 × 10^6^ U/l of penicillin, 100 mg/l of streptomycin and 250 µg/l of amphotericin B at a cell concentration 3x10^5^ cells/ml. A sample of 100 μl of the cell suspension (10^6^ cells/ml) was adhered in 96-well flat-bottomed culture plates for 1 hour at 37 °C. Then 100 μl of serially diluted (protein concentration range 0 - 1.5 mg/ml) coelomic fluid were added into each well and incubated for 18 hours at 37 °C. Cells were then fixed and stained using 100 μl of a 0.5% solution of crystal violet dissolved in 22% ethanol and 8% formaldehyde for 10 min at room temperature. The plates were rinsed in water, 100 μl of 30% acetic acid were added and dye uptake was measured at 620 nm using Microplate reader EL800 (BioTek). The percentage of the lysis was subsequently determined using linear regression. The cytolytic assay was done in duplicates and repeated in three independent experiments.

### Protease assay

To evaluate the protease activity of the coelomic fluid, QuantiCleave™ protease assay kit (Pierce) was used according to the manufacturer´s protocol. Fifty μl of the coelomic fluid diluted in PBS v/v 1:1000, 1:10000 and 1:100000 were incubated at 37 °C for 20 min. Afterwards, 50 μl of QuantiCleave working solution were added to each well and the plate was incubated for 20 min at room temperature. Absorbance at 450 nm was measured using Microplate reader EL800 (BioTek) and the change of absorbance ∆A_450_ for each sample was calculated, where ∆A_450_ is generated by the proteolytic activity of the protease. Calibration curve was prepared from solutions of trypsin in concentration range 0 - 0.5 mg/ml. Standard curve was plotted using logarithmic scale and used to assess relative protease activity of the samples. The assay was performed in duplicates and repeated in three independent experiments.

### Lysoplate assay

To evaluate the lysozyme activity a lysoplate assay was performed according to a modified protocol by Lie [19]. A solution of 1% agarose in 50 mM phosphate buffer (pH 6.0) containing 1 mg/ml of lyophilized *Micrococcus lysodeicticus* (Sigma) was prepared. Samples of *E. andrei* or *E. fetida* coelomic fluids were serially diluted in PBS with final protein concentrations 10, 5, 2.5 and 1.25 mg/ml). Five μl of each sample as well as 5 μl of standard (5 mg/ml hen egg white lysozyme; Roche) were placed on Petri dish and incubated at 37 °C. The diameter of lysed zone (mm) was measured after 24 hours. The assay was done in duplicates and repeated in three independent experiments.

### PCR and real-time PCR using universal primers for fetidin/lysenins genes

To determine the levels of fetidin/lysenins genes in DNA and mRNA universal primers were designed. For designing universal primers, subsequent sequences were chosen: *Eisenia fetida andrei* hemolysin gene (fetidin) (NCBI: U02710), *Eisenia fetida* (*andrei*) mRNA for lysenin (NCBI: D85846), *Eisenia fetida* mRNA for lysenin-related protein 1 (NCBI: D85848), *Eisenia fetida* mRNA for lysenin-related protein 2 (NCBI: D85847), *Eisenia fetida* lysenin-related protein 3 (NCBI: DQ144453). Homologous regions of these sequences were used for determining sets of primers. Primers for both *E. andrei* and *E. fetida* fetidin/lysenins genes used in the PCR and real-time PCR are shown in the Table 2. Forty ng of cDNA and gDNA samples isolated from both species as described above were used for PCR and for iQ^TM^ SYBR® green real-time PCR assay (Biorad).

**Table 2:** Sequences of primers used in real-time PCR experiments.

**Real-time PCR primers**	
**RPL17**	
*sense*	5‘-GCAGAATTCAAGGGACTGGA-3‘
*antisense*	5‘-CTCCTTCTCGGACAGGATGA-3‘
**CCF**	
*sense*	5‘-CTTCACCGACTGGGATCAAT -3‘
*antisense*	5‘-CGTTGTTGTCCGTATTCGTG -3‘
**lysozyme**	
*sense*	5‘-GCCATTCCAAATCAAGGAAC-3‘
*antisense*	5‘-TAGGTACCGTAGCGCTTCAT-3‘
**fetidin/lysenins**	
*sense*	5‘-TGGCCAGCTGCAACTCTT-3‘
*antisense*	5‘-CCAGCGCTGTTTCGGATTAT-3‘

A fragment of 177 bp was amplified by PCR using the following cycling parameters: 2 min at 94 °C, followed by 35 cycles of 30 s at 94 °C, 40 s at 59 °C and 60 s at 72 °C and a final extension for 10 min at 72 °C. The PCR product was analyzed by electrophoresis. 

Differences in gDNA and mRNA levels of fetidin/lysenins genes in both species were determined by using the iCycler^TM^ iQ5^TM^ real-time PCR detection system (Bio-Rad). SYBR GREEN I dye as a fluorescent marker was used. Reaction mixture was perfomed in a volume of 25 μl containing 4 μl of cDNA or gDNA (10 ng/μl), 12.5 μl of SYBR Green Supermix (Biorad) and 0.2 μl of each primer. The setup of reaction in real-time PCR experiment was as follows: 3 min at 95 °C, 40 cycles of 94 °C for 30 s, 59 °C for 40 s, and 72 °C for 70 s. The specificity and efficiency of primer pair was confirmed by melt curve analysis. Differences of gDNA levels were determined as a fold change relative to level of gDNA in *E. fetida*. Change in mRNA expression was evaluated as a fold change relative to the mRNA expression in *E. fetida*. Reference gene RPL17 as an internal control was chosen.

### Determination of cultivable bacteria numbers in compost and in forest soil; isolation and identification of bacteria

Dilution plate cultivation technique was used to estimate the number of bacterial colony forming units (CFU). Triplicate forest soil or compost samples (5 g wet mass) were suspended in 45 ml of 0.2% solution of hexasodium hexametaphosphate, homogenized in an ultrasonic bath (50 kHz, 4 min). Samples were serially diluted (10^-4^ - 10^-7^) and plated (0.2 ml) in quadruplicate onto Trypticase^TM^ Soy Broth agar (TSBA, Becton Dickinson, USA); plates were incubated in the dark at 28 °C for 7 days. Only plates containing 30 to 300 colonies were enumerated, the counts were expressed as CFU/g dry matter of samples. Kruskal−Wallis test (p < 0.05) was used to detect the differences between CFU counts of forest soil and compost (STATISTICA 7). Sixteen isolates of bacterial mixture culture (colonies that differed in morphology) of forest soil and 16 isolates of compost were isolated and identified based on 16S rRNA genes amplification and sequencing according to Kyselková et al. [20]. Briefly, prior to PCR amplification, cell lysates were prepared. One bacteriological loop of bacterial biomass grown on an agar plate was resuspended in 100 μL ultra-pure water. The suspensions were then boiled three times (water bath, 95 °C) for 5 min and frozen at −20 °C for 30 min. The lysates were stored at −20 °C and 1 μL of the lysates was used as a template for PCR.

The identification of isolates were done with 16S rRNA gene amplification using universal bacterial primers [21] pA (5'-AGAGTTTGATCCTGGCTCAG-3') and pH (5'-AAGGAGGTGATCCAGCCGCA-3'), and sequencing. The total volume of PCR reactions was 25 μL. The final reaction mixtures contained (final concentrations) Taq Buffer with (NH_4_)_2_SO_4_ #B33 (Fermentas, 10×), dNTPs (Fermentas, 0.3 mM each), primers (10 µM each) and Dream Taq DNA polymerase (Fermentas; 0.05 U μL^−1^). Bacterial lysate (1 μL, see above) served as a template. Thermal cycling was performed as follows: Initial denaturation at 95 °C for 5 minutes; followed by 34 cycles of denaturation (94 °C/60s), annealing (61 °C/30s) and extension (72 °C/90s) and final extension at 72 °C for 5 min. Amplified 16S rRNA genes were cleaned-up with the GenElute PCR Clean-Up Kit (Sigma) and sequenced (Sanger sequencing, ABI PRISM 3130xl) using the primer pA [21]. The obtained 16S rRNA gene sequences were edited by Bioedit 7.0.4.1 software [22] and Sequence Scanner v 1.0 and assembled using EZ taxon Server 2.1 [23] and database BLAST (Basic local alignment search tool). 

### Cross-colonization experiment

In order to assess the change of expression of selected genes, the experiment with replacement of microbiota environment was performed. Earthworms were maintained on wet paper towels for two days and then transferred to a sterile Petri dish with paper towels soaked with a 10x Antibiotic Antimycotic solution (Sigma) in PBS for decontamination for one day. Cultures of bacteria isolated from the forest soil and from the compost were cultivated in LB broth at 28 °C, subsequently diluted to 10^8^ CFU/ml in PBS and the suspension was used for microbial stimulation. Earthworms were then maintained on paper soaked with bacterial suspension for seven days. Coelomocytes were harvested in an interval of one day, three days and seven days after the stimulation. Coelomocytes from earthworms maintained in bacterial mixture originated from their natural environment were used as a negative control. RNA from coelomocytes was isolated, reverse transcribed and cDNA was used in real-time PCR analysis to determine differences in the expression for CCF, fetidin/lysenins and lysozyme genes. Specific primers used in real-time PCR are summarized in the Table 2.

### Statistics

Data were expressed as a mean ± SD of the values obtained from three independent experiments performed in duplicates. One-way ANOVA with Dunnett's post test was performed using GraphPad Prism software to evaluate the significance of the data. Differences were considered significant when P < 0.05.

## Results

### Cytochrome *c* oxidase I gene as a potential barcode to distinguish related species

**Figure 1:**
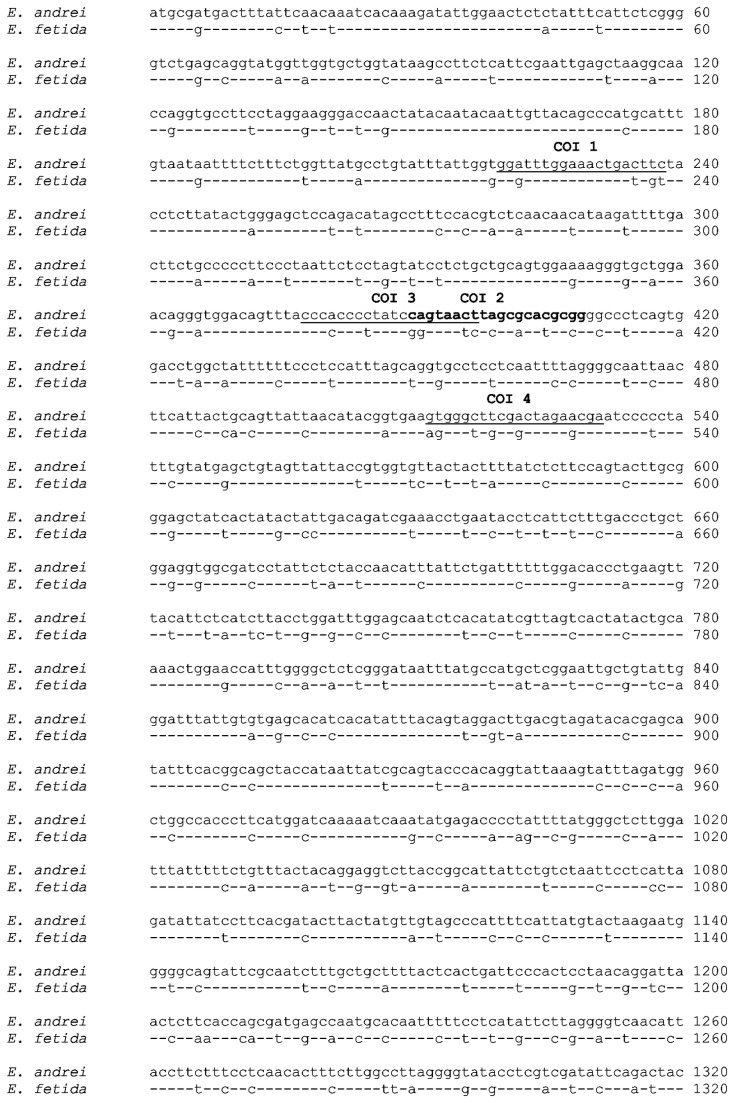
Alignment of *E. andrei* and *E. fetida* COI sequences. Alignment of *E. andrei* and *E. fetida* COI sequences. Oligonucleotides used as discriminating primers for *E. andrei* or *E. fetida* COI are underlined or in bold (refer to the Table 1).

Molecular differentiation on a basis of polymorphism of mitochondrial gene for COI is widely used for discrimination of closely related animal species. It was previously published that nucleotide sequences of COI differ between *E. andrei* and *E. fetida* species [15]. Based on these published sequences, pairs of primers specific for both *E. andrei* and *E. fetida* COI were designed. Using these primer pairs with *E. andrei* or *E. fetida* cDNA as a template in PCR reaction, fragments of COI of 541 bp were obtained and subsequently sequenced. Alignment of these two sequences confirmed the presence of single- or double-nucleotide mismatches dispersed all along these sequences. In order to obtain the full-length sequences of *E. andrei* and *E. fetida* COI genes, RACE amplifications of 5’ as well as 3’ ends were performed. Resulting PCR products were cloned and sequenced. Consequently, the whole coding sequences of *E. andrei* and *E. fetida* COI with open reading frames of 1542 bp encoding 514 amino acids were obtained (Figure 1) and submitted to the GenBank databasis (*E. andrei* COI - NCBI: HQ534065, *E. fetida* COI - NCBI: HQ534066). The alignment of both sequences revealed a high level of homology (80%).

**Figure 2:**
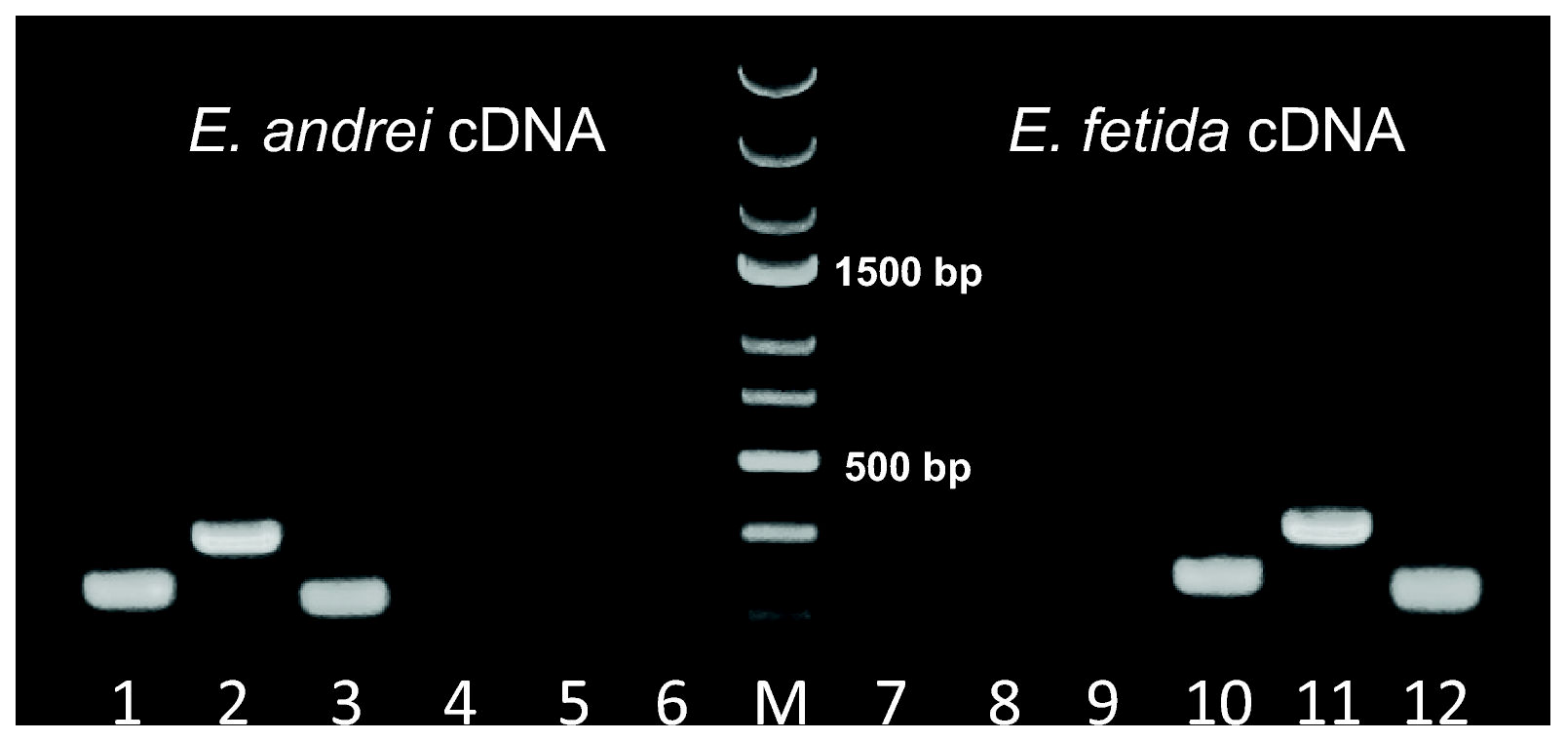
Species-specific PCR discriminating COI genes. Species-specific PCR. Lanes 1-6: *E. andrei* cDNA, lanes 7-12: *E. fetida* cDNA. *E. andrei* specific primers combinations: lane 1 and 7: COI EA 1/COI EA 2; lane 2 and 8: COI EA 1/COI EA 4; lane 3 and 9: COI EA 3/COI EA 4. *E. fetida* specific primers combinations: lane 4 and 10: COI EF 1/COI EF 2; lane 5 and 11: COI EF 1/COI EF 4; lane 6 and 12: COI EF 3/COI EF 4. DNA ladder marker (M) is in the middle (GeneRuler Express DNA Ladder, Fermentas).

Taking advantage of minor differences in *E. andrei* and *E. fetida* COI sequences, sets of primers discriminating between the two species were designed (Table 1, Figure 1) and used in a PCR reaction with stringent conditions, i.e. a high annealing temperature and decreased amount of cycles. By using primers specific for *E. andrei* COI, we could detect PCR products only in reactions containing *E. andrei* cDNA while no PCR product was detected if *E. fetida* cDNA was used as a template. Conversely, primers specific for *E. fetida* COI binds solely to *E. fetida* cDNA and not to *E. andrei* cDNA (Figure 2). Therefore, these primers can be used as a reliable tool for the differentiation of these two species.

### Biological activities of the coelomic fluid

In order to compare the immunological properties of the coelomic fluid of *E. andrei* and *E. fetida* various bioassays were performed. For the evaluation of the coelomic-fluid hemolytic activity, a hemolytic assay using sheep erythrocytes was performed. Samples of CF of *E. andrei* or *E. fetida* in protein concentration range 0 - 100 μg/ml with inhibitors of serine proteases that eliminate unspecific hemolytic activity, was combined with 3% erythrocyte suspension. CF of *E. andrei* exhibits stronger hemolytic activity when compared to CF of *E. fetida*. CF of *E. andrei* causes 50% hemolysis when diluted to 1.9 μg/ml whereas CF of *E. fetida* causes the same level of hemolysis at the concentration higher than 50 μg/ml (Table 3A).

**Table 3:** Biological activities of coelomic fluids.

	**A** hemolytic activity	**B** cytolytic activity	**C** protease activity
*E. andrei*	1,9 μg/ml	133,9 μg/ml	2000 μg/ml
*E. fetida*	52,2 μg/ml	128,2 μg/ml	1700 μg/ml

Comparison of hemolytic activity (A), cytolytic activity (B) and protease activity (C) of both species.

A: Hemolytic activity value represents the CF concentration needed for lysis of 50% of erythrocytes.

B: Cytolytic activity of coelomic fluid was measured as lysis of L929 fibroblasts. Values represent concentrations of the coelomic fluid required for lysis 50% of fibroblasts.

C: Relative protease activity of *E. andrei* and *E. fetida* coelomic fluid was calculated from the samples of coelomic fluid diluted 1/10000 in H_2_O. Protease activity is expressed as an equivalent of trypsin standard activity.

Cytolytic activity was determined as a percentage of L929 fibrosarcoma cell lysis depending on the concentration of coelomic fluid. CF of both earthworm species showed a strong cytolytic activity. Almost 100% of lysis was observed when cells were treated with CF at the concentration 600 µg/ml or higher. Protein concentration necessary for 50% of lysis was very similar in both species (Table 3B).

Relative protease level of *E. andrei* and *E. fetida* CF was calculated from the samples diluted 1/10000 in H_2_O. Using the standard logarithmic curve, the concentration of proteases of CF of *E. andrei* was determined to be 2.0 mg/ml while the protease concentration of CF of *E. fetida* was calculated as 1.7 mg/ml (Table 3C).

**Figure 3:**
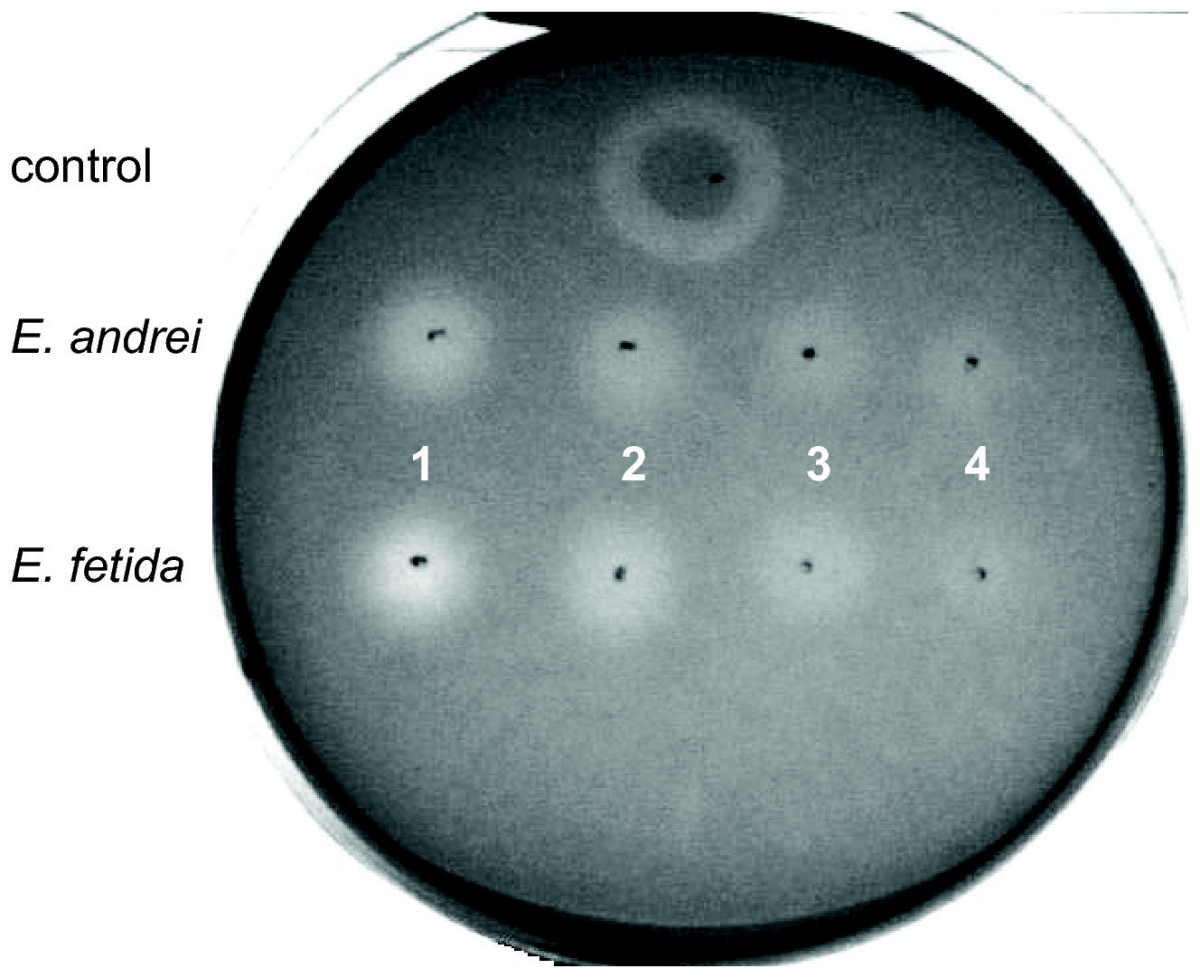
Lysoplate assay. Lysozyme activity of coelomic fluid isolated from *E. andrei* or *E. fetida* was evaluated by measurement of the diameters of the cleared zones (representative data of one of three independent experiments). As positive control hen egg white lysozyme (25 µg) was used. Lysozyme activity of coelomic fluid with total protein amount of (1) 50 µg, (2) 25 µg, (3) 12.5 µg and (4) 6.25 µg was measured.

The lysozyme activity in a CF of *E. andrei* and *E. fetida* was evaluated qualitatively using a lysoplate assay (Figure 3). Samples of *E. andrei* and *E. fetida* CF with total protein amount of 50 μg (1), 25 μg (2), 12.5 μg (3) and 6.25 μg (4) were incubated with *Micrococcus lysodeicticus* in 1% agarose to form lyzed zones. The diameter of the control lysed zone was 18 mm. The diameters of zones cleared by *E. andrei* coelomic fluid were (1) 9 mm, (2) 7 mm and (3) 5 mm, whereas zones lysed by coelomic fluid isolated from *E. fetida* had diameters of (1) 10 mm, (2) 8 mm and (3) 5 mm, respectively. There was no measurable cleared zone when 6.25 µg of total coelomic fluid proteins were used. We did not find any differences in the lysozyme activity of both species.

### Fetidin/lysenins genes and their expression

**Figure 4:**
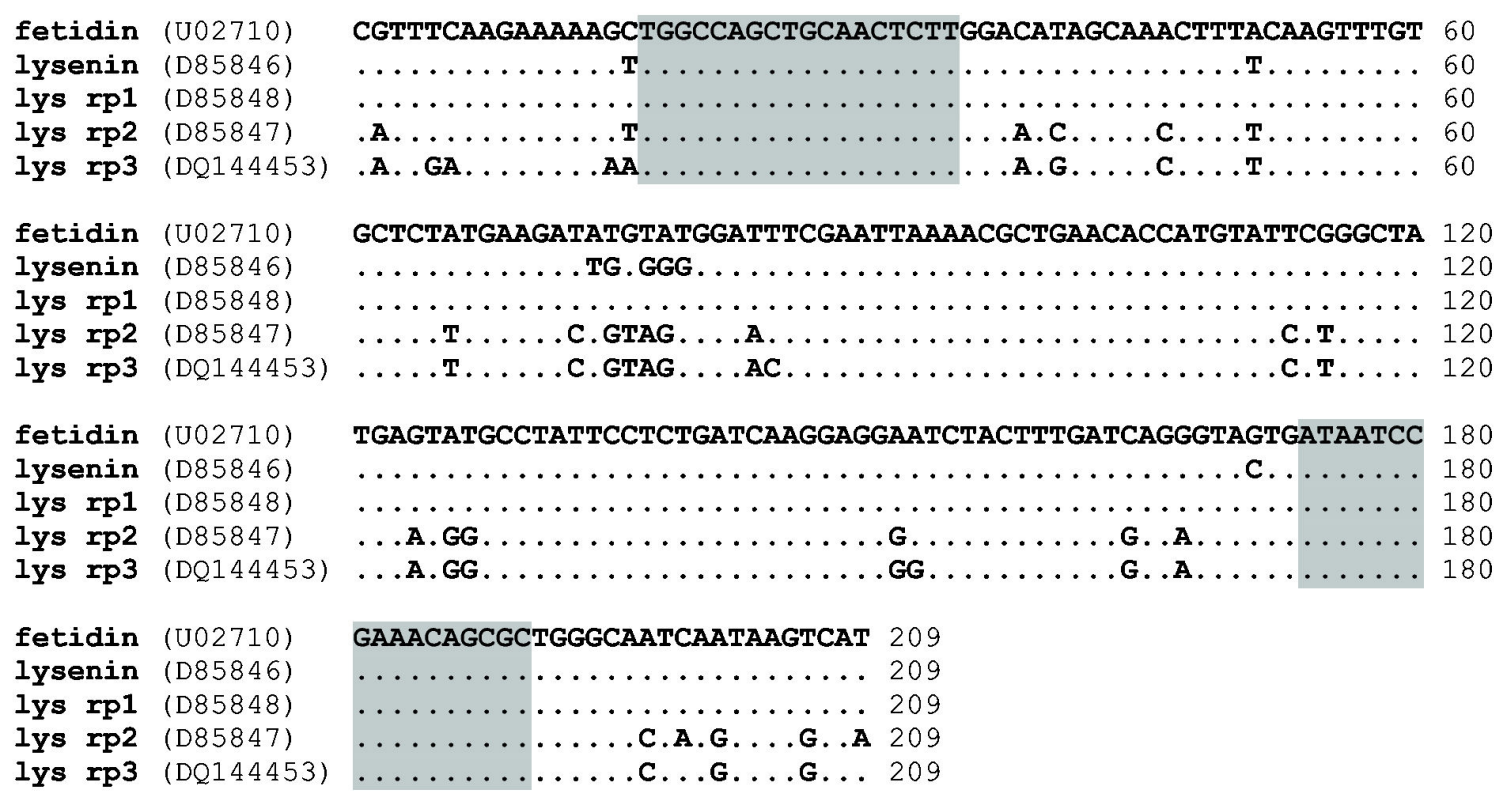
Fetidin-lysenins genes alignment. The alignment of partial sequences of fetidin-lysenins genes. Universal primers (highlighted) were designed on the basis of presence of homologous regions.

**Figure 5:**
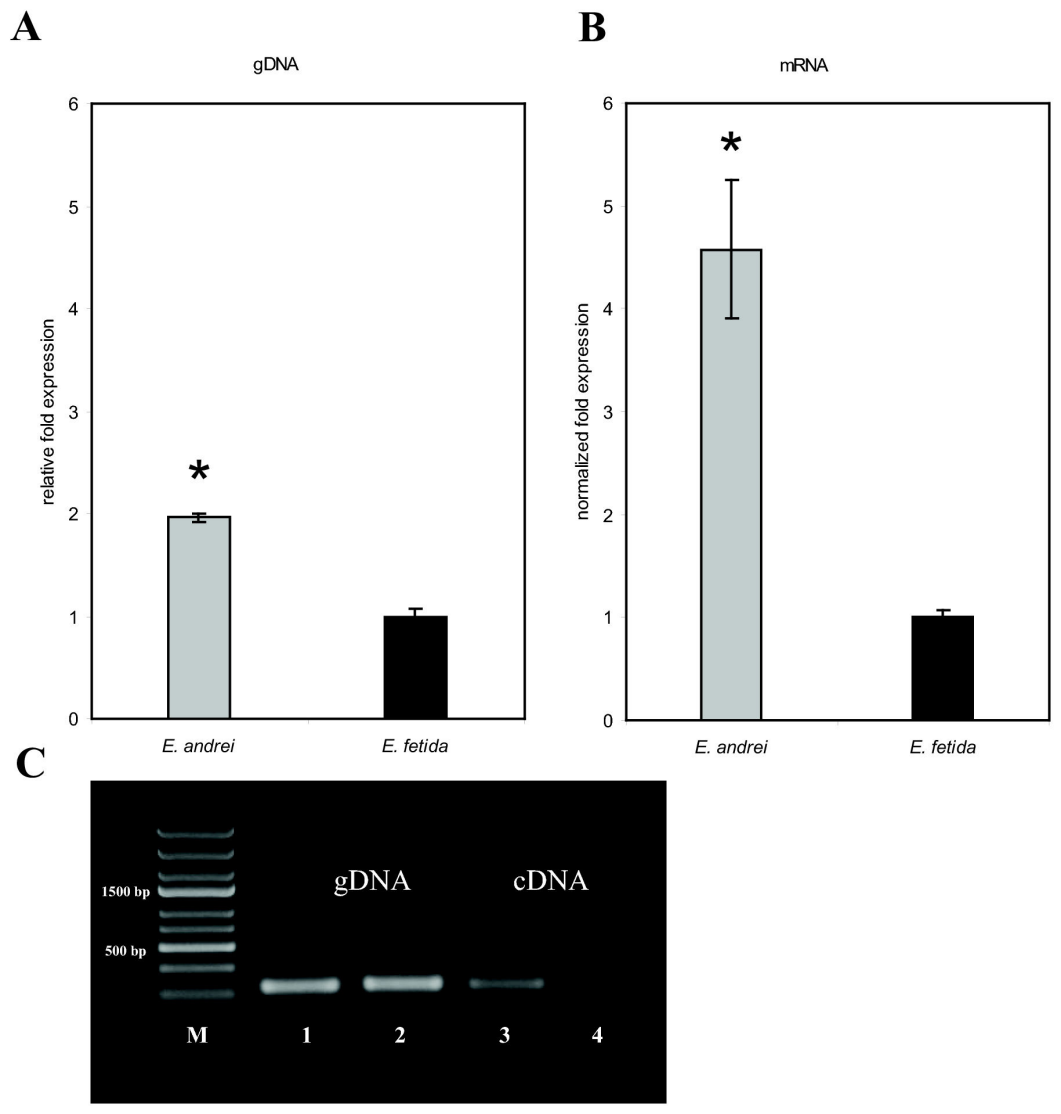
gDNA and mRNA levels of fetidin/lysenins genes. gDNA and mRNA levels of fetidin/lysenins genes in *E. andrei* and *E. fetida* determined by real-time PCR and PCR. The values are the means of three independent experiments (± SD) performed in duplicates (*P < 0.05). **A**: gDNA levels of fetidin/lysenins genes relative to *E. fetida*. **B**: Fold change in gene expression relative to expression of fetidin/lysenins in *E. fetida*. Gene expression was normalized for the reference gene RPL 17 (ribosomal protein L17). **C**: PCR analysis using primers for lysenin genes reveals the presence of these genes in genomic DNA and mRNA of both species. Genomic DNA and cDNA was amplified using universal primers for fetidin/lysenins. Lane 1, 3: *E. andrei* Lane 2, 4 *E. fetida*. DNA marker (M) is on the left margin (GeneRuler Express DNA Ladder, Fermentas).

In order to analyze the expression of all genes related to fetidin and lysenins, we intended to use primers encompassing all cognate sequences in real-time PCR analysis. The alignment of selected sequences of fetidin/lysenins genes is shown in Figure 4. Conserved DNA segments longer than 20 nucleotides were selected for design of a suitable primer pair. Levels of mRNAs and gDNA encoding for defense factors fetidin/lysenins in *E. andrei* and *E. fetida* were compared. The specificity and efficiency of universal primers was confirmed by melting curve analysis and was approximately 96%. The level of fetidin/lysenins genes amplified by real-time PCR was twice as high in *E. andrei* as in *E. fetida* (Figure 5A). In the case of mRNA levels the expression was five times higher in *E. andrei* than in *E. fetida* (Figure 5B). Results from quantitative real-time PCR assay are in accordance with PCR. One particular band was detected in all samples with using gel electrophoresis (Figure 5C).

### Bacterial characterization of earthworm habitats

Cultivation analysis indicated that mixtures of bacterial cultures of forest soil and compost samples differed in CFU counts as well as composition of isolates. Compost contained significantly higher numbers of culturable forms of bacteria than the forest soil; the difference represented two orders of magnitude (Table 4). The 16S rRNA analysis of isolates indicated that Gram-positive bacteria dominated in compost sample (Table 5). Nine isolates represented class Actinobacteria, two isolates belonged to the class of Bacilli. Two isolates were not identified, only three isolates belonged to Gram-negative bacteria, one isolate to class Alpha Proteobacteria and two to Gamma Proteobacteria. Representation of Gram-positive and Gram-negative bacteria in forest soil was in equilibrium, six isolates belonged to the class of Bacilli, two to Actinobacteria seven to Gamma Proteobacteria and one isolate to the class of Beta Proteobacteria. 

**Table 4:** Numbers of bacteria in forest soil and in compost.

	**CFU . g^-1^ dry substrate**
forest soil (*E. fetida*)	0.65 ± 0.17 . 10^6^
compost (*E. andrei*)	115.85 ± 8.23 . 10^6^

The numbers of culturable forms of bacteria in a sample of forest soil and compost.

**Table 5:** Bacterial composition in forest soil and in compost.

**Forest soil bacterial isolates**			
**Isol.**	**Closest database reference**	**GenBank acc. no.**	**Similarity %**
**F1**	*N.A.*		
**F2**	*Bacillus licheniformis ATCC 14580(T)*	AE017333	99.77
**F3**	*Bacillus mycoides DSM 2048(T)*	ACMU01000002	100.00
**F4**	*Arthrobacter humicola* KV-653(*T*)	AB279890	99.87
**F5**	*Pseudomonas kilonensis 520-20(T)*	AJ292426	98.93
**F6**	*Pseudomonas putida DSM 291(T)*	Z76667	98.28
**F7**	*Bacillus weihenstephanensis WSBC10204(T)*	Z84578	99.78
**F8**	*Pseudomonas fragi ATCC 4973(T)*	AF094733	100.00
**F9**	*Pseudomonas baetica a390(T)*	FM201274	99.75
**F10**	*Viridibacillus arenosi LMG 22166(T)*	AJ627212	100.00
**F11**	*Bacillus simplex NBRC 15720(T)*	AB363738	100.00
**F12**	*Pseudomonas jessenii CIP 105274(T)*	AF068259	99.07
**F13**	*N.A.*		
**F14**	*N.A.*		
**F15**	*Arthrobacter antarcticus SPC26(T)*	AM931709	98.53
**F16**	*Sporosarcina globispora DSM 4(T)*	X68415	98.08
**Compost bacterial isolates**			
**C1**	*Rhodanobacter fulvus Jip2(T)*	AB100608	99.28
**C2**	*Microbacterium profundi Shh49(T)*	EF623999	98.04
**C3**	*Microbacterium natoriense TNJL143-2(T)*	AY566291	99.08
**C4**	*Microbacterium ketosireducens IFO 14548(T)*	AB004724	97.94
**C5**	*Microbacterium natoriense TNJL143-2(T)*	AY566291	100.00
**C6**	*N.A.*		
**C7**	*N.A.*		
**C8**	*Staphylococcus epidermidis ATCC 14990(T)*	L37605	100.00
**C9**	*Microbacterium ulmi XIL02(T)*	AY062021	97.87
**C10**	*Microbacterium lacus A5E-52(T)*	AB286030	99.05
**C11**	*Microbacterium ulmi XIL02(T)*	AY062021	99.14
**C12**	*Streptomyces griseoaurantiacus NBRC 15440(T)*	AB184676	97.84
**C13**	*Micrococcus luteus NCTC 2665(T)*	CP001628	100.00
**C14**	*Asticcacaulis benevestitus Z-0023(T)*	NR_042433	99.74
**C16**	*Bacillus stratosphericus 41KF2a(T)*	AJ831841	100.00

List of bacterial isolates, identification by 16S rRNA gene sequence analysis; % - similarity to the closest type strain in EZ taxon Server 2.1 and database BLAST; *N.A. – not analyzed.*

### Cross-colonization

**Figure 6:**
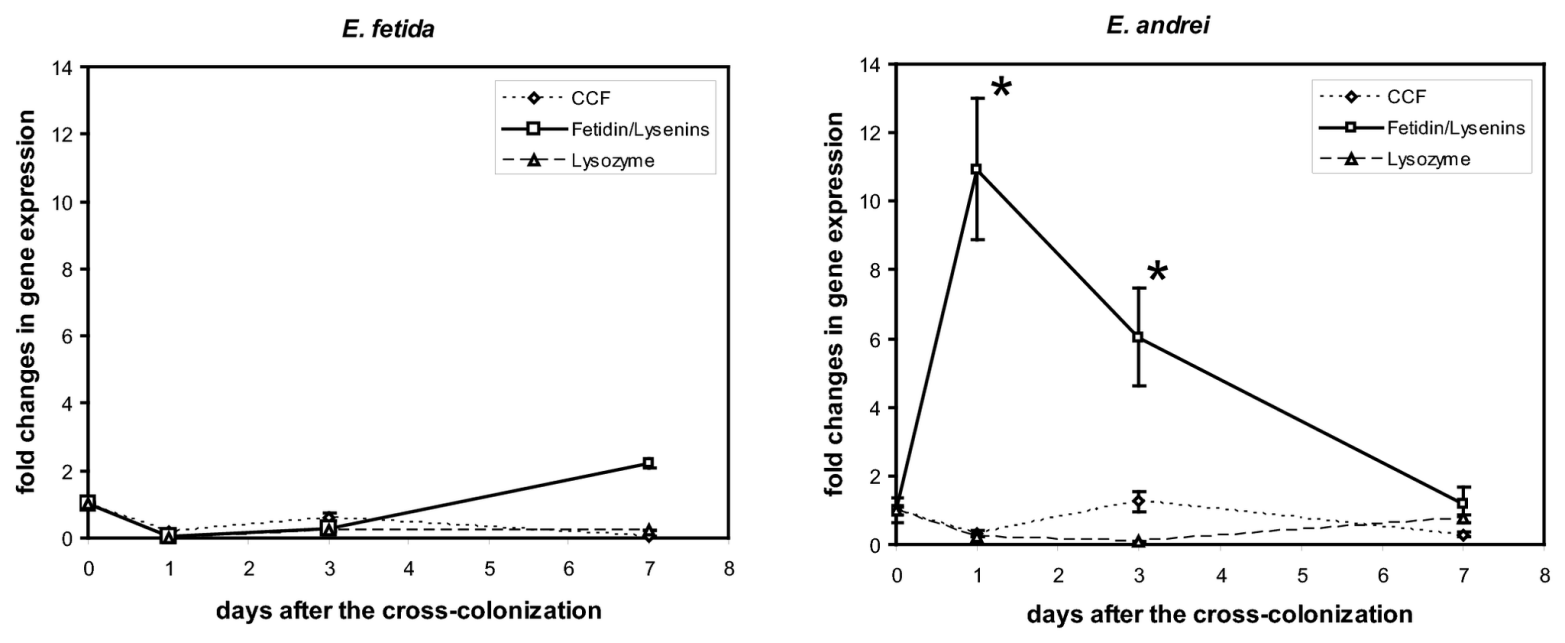
Comparison of gene expression levels in *E. andrei* and *E. fetida*. Gene expression levels of selected genes (CCF, lysozyme, fetidin/lysenins genes) in *E. andrei* and *E. fetida* earthworms upon bacterial cross-colonization determined by real-time PCR and normalized for the reference gene RPL17 (ribosomal protein L17). Fold change in the gene expression are relative to the expression in earthworms maintained with bacteria isolated from their natural environment. The values are the means of three independent experiments (± SD) performed in duplicates (* P < 0.05).

To observe the influence of microbiota on the expression of selected genes (CCF, lysozyme, fetidin/lysenis) cross-colonization experiments were performed. The gene expression analysis has revealed an increase of fetidin/lysenins in *E. andrei*, while the expression of the same genes displays only minimal changes in *E. fetida*. Changes in the gene expression of CCF and lysozyme are not significant in both species (Figure 6).

## Discussion

The soil naturally consists of layers with different composition of mineral and biological materials that determine ecological niches for soil organisms and their survival in certain part of the soil. Accordingly, earthworms can be divided into three groups, called ecotypes reflecting different segments of the soil horizon. Top layer of the soil is rich in decaying organic matter and it is characterized by a high variability of microbiota. Earthworms living in this environment belong to the epigeic species (e.g. *Dendrobaena octaedra, Eisenia andrei, Eisenia fetida, Lumbricus rubellus*). Endogeic earthworms are found under the topsoil. This environment is characterized by a lower amount of organic residues and by decreasing variability of microbiota. Among endogeic earthworms belong species like *Aporrectodea caliginosa, Aporrectodea rosea, Octolasion lacteum*. Anecic earthworms (e.g. *Aporrectodea longa*, *Fitzingeria platyura*, *Lumbricus terrestris*) live in burrows in deep mineral soil layers characterized by the lowest microbial load, but come to the surface to feed on dead leaves, which they drag into their burrows. Previously we focused on a study of pattern recognition molecule CCF in earthworms belonging into these three ecotypes. CCF of *Eisenia* has a broader saccharide-binding specificity in comparison with other earthworm species [11]. *Eisenia* as an epigeic earthworm needs to be resistant against various microorganisms present in the top layer of the soil. Earthworms living in the lower soil horizons are exposed to a weaker antigenic pressure and their CCF possesses a limited pattern recognition capacity. More variable and potent binding capacity of *Eisenia* CCF assumes a better tool for the recognition of potential pathogenic bacteria. Heterogeneity of microbiota represents a higher pressure to the immune system of earthworms and thus we can hypothesize that the microbial environment can play a crucial role for the development of defense system of earthworms.

Based on this assumption we focused on the comparison of defense system of two closely related epigeic earthworms, *E. andrei* and *E. fetida*. These two earthworms share many physiological properties but their natural environment distinctly differs that can affect their immune system.

The taxonomy of *E. andrei*/*E. fetida* is complicated since the most of current literature uses indiscriminately the term *E. fetida* and often it is not clear, which of the two species is being referred to. Mitochondrial COI gene is widely used as a DNA barcode for the identification of animal samples. Peréz-Losada et al. have determined these two species based on mitochondrial and nuclear DNA sequences using conserved primers amplifying COI fragments of most species [15], while we designed and used discrimination primers specific only for one species. Differences in COI sequences of both species are distributed in the entire length of obtained sequences (Figure 1), therefore we were able to design suitable sets of primer pairs. The main advantage of such species-specific primer pairs is the possibility to quickly discriminate *E. andrei* and *E. fetida* without the requirement of sequencing. 

As described previously, the coelomic fluid exhibits many biological activities involved in the innate defense of earthworms. Approximately 40% of the cytolytic activity of CF is caused by the pattern-recognition molecule CCF [8]. Sequences of CCF of *E. andrei* and *E. fetida* were obtained and the alignment did not show any significant differences in the amino acid sequence (data not shown). This close similarity of CCF molecules is in accordance with the minimal differences in the cytolytic activity of the coelomic fluids. Lysozyme-like activity is another antimicrobial property of the earthworm coelomic fluid [4,24]. We assessed the lysozyme-like activity in the coelomic fluids of both species and no differences in the activity were observed. Moreover, the sequence of *E. fetida* lysozyme gene was obtained and aligned with previously described sequence of *E. andrei* and the alignment of both sequences showed a high level of homology of these molecules (data not shown). Similarly, we did not observe any significant differences in the proteolytic activity of the coelomic fluid of *E. andrei* and *E. fetida* that could affect a proper prophenoloxidase cascade activation [25] or other immunodefense pathways [26]. It should be noted that microorganisms form a considerable part of the earthworm diet [27] and thus, proteases and lysozyme play an important role as digestive/nutritional enzymes in the gut [28]. However, we follow protease and lysozyme activities in the coelomic fluid suggesting rather their defense function. Taken together, the above mentioned biological activities assessed in the coelomic fluid of both species are very similar and accordingly, the primary structures of the effector molecules (CCF and lysozyme) are highly homologous.

However, antibacterial activity of the coelomic fluid is mediated by various proteins. Interestingly, some of these proteins cause hemolysis of various erythrocytes of vertebrates. This hemolytic activity was first described by Du Pasquier [29] and later on, some of these proteins were characterized at the molecular level [6,7,30], nevertheless a final classification of all hemolytic proteins remains unresolved. In 2006, the presence of two distinct genes with a high level of homology coding for fetidin and lysenin was documented (Procházková et al. 2006). The presence of DNA coding for both proteins at the genomic and cDNA levels was observed in all tested earthworms suggesting that fetidin and lysenin do not result from posttranscriptional splicing or other modifications of the transcript. Since *Eisenia* earthworms are considered as diploid animals with 22 chromosomes [31,32], the possibility that both proteins are encoded by different alleles of one gene is not probable because all tested individuals would have to be heterozygotes. 

Here we show that the hemolytic activity of *E. andrei* coelomic fluid is much higher as compared to that one of *E. fetida*. Differences in the hemolytic activity of the coelomic fluid of both species led us to search for some possibility of the quantification of hemolytic factors. High variability of hemolytic patterns and differences in the expression of fetidin and lysenins in *Eisenia* were previously observed [33]. From that reason we designed a universal primer pair for the detection of all known fetidin- and lysenins-related molecules with the hemolytic activity. Quantitative real-time PCR confirmed differences between these two species at the level of genomic DNA as well as mRNA. Valembois et al. [34] described a system of hemolytic families based on the natural polymorphism. Hemolytic phenotype of each individual consists of one protein of pI 6.0 and of the second protein that may be present in a form of four possible alleles. One of these four alleles was detected only in the population of earthworms harvested in industrial vermicompost and originating from California and never was found in European population of earthworms. The presence of the homozygous allele b confers an important defense advantage toward pathogenic bacterial infestation [35]. Our results showing twice higher number of fetidin/lysenins gene copies in the genomic DNA may suggest that one or more of these genes were duplicated/multiplicated in the genome of *E. andrei*. Since we used a universal primer pair for the detection of all known fetidin and lysenins-related genes our assumption remains hypothetical.

There was substantially higher quantity of bacteria in compost as compared to the soil. Therefore a question has arisen whether the change of the microbial environment can influence the expression of defense molecules. As it was described previously, the defense system of earthworms can be stimulated by the microbial challenge [4,10]. In order to monitor the possible adaptation of earthworms to an unknown microbiota, the cross-colonization experiments were performed. Initially, we planned to determine the reaction of earthworms after the replacement of their natural environment. *E. andrei* earthworms were placed to the forest soil and *vice versa*, *E. fetida* earthworms to the compost. *E. fetida* earthworms appeared to be very sensitive to low pH of the compost and did not survive for more than two or three days (data not shown). Therefore, we isolated bacterial strains from both compost and forest soil, cultured them and the mixtures were used for the stimulation in the cross-colonization experiments. While the expression of fetidin/lysenins was significantly upregulated in *E. andrei*, biologically non-significant changes were found in the case of *E. fetida* challenged with compost microbiota. The absence of detectable reaction of *E. fetida* to compost microbiota can be explained either by the lower number of gene copies coding for fetidin/lysenins as compared to *E. andrei* or by unknown difference of the gene expression regulation in both species.

In summary, we demonstrated the effect of compost and forest-soil microbiota on the immune mechanisms of *E. fetida* and *E. andrei* earthworms. While the gene expression and biological activities of lysozyme and CCF do not differ in both species, the gene expression of fetidin and lysenin genes as well as the hemolytic activity of the coelomic fluid of *E. andrei* is significantly higher in comparison with that one of *E. fetida*. Genomic DNA analyses revealed approximately twice higher number of fetidin/lysenins gene copies in *E. andrei* as compared to *E. fetida*. It can be hypothesized that *E. andrei* colonizing compost as a new habitat acquired an evolutionary selection advantage resulting in a higher expression of antimicrobial proteins. 
